# Effects of Blackcurrant Extract During High-Intensity Intermittent Running: An Exploratory Study of Possible Muscle Fibre-Type Dependence

**DOI:** 10.3390/muscles4040056

**Published:** 2025-11-14

**Authors:** Mark E. T. Willems, Sam D. Blacker, Ian C. Perkins

**Affiliations:** School of Sport, Science and Engineering, University of Chichester, Chichester PO19 6PE, UK; s.blacker@chi.ac.uk (S.D.B.); i.perkins@chi.ac.uk (I.C.P.)

**Keywords:** anthocyanins, fatigue, high-intensity exercise, muscle fibre type, antioxidant, treadmill running, polyphenols, exercise physiology, muscle physiology

## Abstract

Intake of anthocyanin-rich blackcurrant extract showed muscle fibre-type specific force responses during fatigue development from combined use of voluntary maximal isometric contractions and electrically evoked twitch contractions of the *m. quadriceps femoris*. In the present exploratory study, we examined the fibre-type specific effects by blackcurrant extract on high-intensity intermittent treadmill running performance to exhaustion. Active males (*n* = 16, age: 23 ± 3 years, height: 179 ± 5 cm, body mass: 79 ± 3 kg, $\dot{V}$O_2max_: 55.3 ± 5.0 mL·kg^−1^·min^−1^) completed a fatiguing protocol with 16 voluntary maximal isometric contractions to predict muscle fibre typology. The high-intensity intermittent running protocol was completed twice following a 7-day intake of blackcurrant extract (210 mg anthocyanins per day) and twice following a placebo (PL) in a randomized, double blind, crossover design. Heart rate and lactate were recorded at exhaustion. Data were averaged for each condition. There were no significant correlations between the percentage force decline by the repeated isometric contractions (mean ± SD: 29.3 ± 12.4%) and total and high-intensity running distance. Participants were categorized into a predominant muscle fibre type I (slow-twitch, *n* = 3 with the lowest isometric force decline: 12 ± 9%) and type II typology (fast-twitch, *n* = 3 with the highest isometric force decline: 46 ± 10%). Only the individuals with a predominant type I fibre typology improved the total running and high-intensity running distance by 17 ± 12% and 15 ± 11%. At exhaustion, there were no differences between individuals with a type I or II fibre typology for heart rate and lactate. These exploratory results suggest that the ergogenic potential of anthocyanin-rich blackcurrant extract on high-intensity intermittent exercise may depend on muscle fibre type, though larger and more robust studies are needed to confirm this observation. Future work will establish whether our exploratory results contributed to our understanding of the underpinning of inter-individual responses to the intake of anthocyanin-rich nutritional ergogenic aids.

## 1. Introduction

In the last decade, the ergogenic potential of anthocyanin-rich blackcurrant has been shown in various exercise models including high-intensity and maximal exercise [1,2,3]. During high-intensity exercise, the recruitment and high activity of type II (fast-twitch) muscle fibres is required [4]. Force production by the type II muscle fibres relies predominantly on the phosphagen and glycolytic energy pathways. However, the associated by-products of these energy pathways can rapidly initiate the onset of peripheral muscle fatigue and reduce muscle function (for a review see [5]). In a study by Lievens et al. [6], proton magnetic resonance spectroscopy was used to categorize male, recreationally active participants with a slow- and fast-twitch typology to examine the outcome of the Wingate cycling test and showed differences. As expected, more fatigue was present in those with a fast-twitch muscle fibre type during the Wingate cycling test [6]. As far as we know, one of the physiological factors that may affect the response to a dietary intervention, i.e., muscle fibre type, has never been used as an inclusion criterion. The ergogenic potential of dietary supplements to enhance high-intensity exercise performance can affect the physiology of specific muscle fibres. For example, dietary organic nitrate-enhanced intracellular calcium handling in mice fast-twitch muscle fibres resulted in an increased force production; this was not observed in slow-twitch muscle fibres [7]. With creatine supplementation in rats, beneficial effects were also shown in fast-twitch muscle fibres [8]. In order to advance our understanding of the performance enhancing effects of the intake of anthocyanin-rich blackcurrant, it is feasible to expose isolated slow- and fast-twitch muscle fibres to anthocyanins and anthocyanin-induced metabolites to examine alteration of cellular function. Due to the very low bioavailability of anthocyanins [9], it is likely that the beneficial effects of anthocyanin intake are occurring due to the numerous and synergistic actions of the anthocyanin-induced metabolites (e.g., hippuric acid [10], isovanillic acid [11], and protocatechuic acid [12,13]) which alter cell function. Many reviews have highlighted the interindividual bioavailability of metabolites and the complex role of the metabolites in altering cell function, human physiology, and health (e.g., [14,15,16]). An indirect approach to examine the potential effects of muscle fibre type on in vivo exercise performance in humans is therefore justified.

In the study by Hamada et al. [17], the individual force declined during repeated voluntary maximal isometric contractions and the quantification of muscle fibre type with muscle biopsy observations [18] allowed for the functional identification of individuals with primarily type I or II muscle fibre typology. The testing procedure of repeated voluntary maximal isometric contractions by Hamada et al. [17] was used in the present study. When the Hamada et al. protocol [17] was used in a previous study on the effects of NZBC extract [19], observations suggested that individuals with primarily slow-twitch muscle fibre typology had increased fatigue resistance based on twitch responses of the *m. quadriceps femoris*. Furthermore, it was observed that during the repeated voluntary isometric contractions, those individuals had higher isometric maximal forces in the final quartile of the exercise protocol [19]. It is not known whether such observations would translate to an understanding of whole-body performance during high-intensity intermittent exercise.

The effectiveness of dietary supplementation to enhance exercise performance (e.g., caffeine, dietary nitrate (beetroot), creatine monohydrate, and β-alanine) can be due to improved energy metabolism in active skeletal muscles as well as reducing or postponing the exercise-induced peripheral muscle fatigue mechanisms (for reviews see [20,21]). For example, intake of creatine monohydrate increases intracellular phosphocreatine content [22] and enhances exercise-required ATP resynthesis. The ergogenic potential created by the intake of creatine monohydrate is mostly observed during repeated high-intensity exercise [23,24]. For beta-alanine supplementation, the ergogenic effect on high-intensity exercise is due to the enhanced intracellular muscle carnosine content which has the subsequent ability to limit the exercise-induced rise in protons in muscle cells [25] and affects the peripheral muscle fatigue mechanisms. In most of these earlier studies, it was not common to report on the inter-individual responses to the intake of nutritional ergogenic aids. It was the recognition of the importance of personalized nutrition [26] that contributed to the need for the unravelling and analysis of the inter-individual responses to nutritional ergogenic aids [27]. The number of supplementation studies that address the inter-individual responses, as well as the consistency by repeated measures, are still limited (e.g., sodium bicarbonate [28], blackcurrant anthocyanins [29]). However, many studies without repeated testing for the effectiveness of the supplement are now addressing the cohort and the interindividual responses (e.g., purple grape juice [30], blueberry [31], vitamin C [32]). Many factors can contribute to the interindividual responses such as genotype (caffeine [33]), sex (sodium bicarbonate [34]), training status (β-alanine [35]), and intake timing (Montmorency cherry [36]). Most times, supplementation studies employ inclusion criteria such as sex and time of day testing, among others, to reduce potential inter-individual variation in the physiological, metabolic, and performance responses. Interestingly, studies that examined sex differences in exercise responses to dietary supplementation (sodium bicarbonate: [34] and caffeine: [37]) interpret the findings due to the differences in muscle fibre types.

Therefore, the aim of the present study was to examine whether the enhanced blackcurrant-induced performance response during high-intensity intermittent treadmill running was related to muscle fibre typology. From a cohort of sixteen male participants, the three participants with the lowest and highest force decline during a series of voluntary maximal isometric contractions were categorized as having predominantly type I or type II muscle fibre typology. Our observations seem to indicate that the ergogenic potential of New Zealand blackcurrant extract for enhancing the performance of repeated high-intensity exercise is muscle fibre-type dependent and suggests an increased fatigue resistance in type I muscle fibres. Our pilot observations may contribute to our understanding of the underpinning of the interindividual responses to high-intensity exercise by the intake of anthocyanin-rich New Zealand blackcurrant extract.

## 2. Results

**Figure 1:**
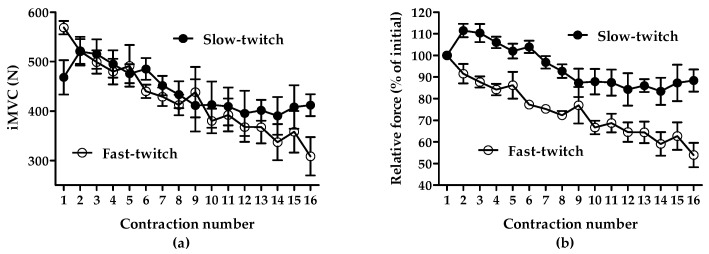
The relationship between absolute force from an iMVC (**a**) and relative force (**b**) as a function of contraction number from a series of 16 isometric maximal voluntary isometric contractions of the *m.quadriceps femoris*. iMVC, isometric maximal voluntary contraction. Data are the mean ± SD from 3 participants identified as having predominant a slow-twitch or fast-twitch muscle fibre typology.

The correlations between the force decline (%) during the 16 repeated voluntary isometric maximal contractions and the change in total running distance (r = −0.31, *p* = 0.246) and high-intensity running distance (r = −0.24, *p* = 0.369) by the intake of NZBC extract were not significant. For the cohort, the force decline (%) during the 16 repeated voluntary isometric contractions was 29.3 ± 12.4% (95%CI [22.6, 35.9%], range: 1.3–56.8%). Based on methodology and observations by Hamada et al. [17], the three participants with the lowest force decline (i.e., 1.3%, 15.9% and 17.4%) and the three participants with the largest force decline (i.e., 37.9%, 43.4% and 56.8%) were classified as having predominantly slow-twitch and fast-twitch muscle fibre typology. Figure 1 shows the absolute (a) and relative force decline (%) (b) as a function of isometric contraction number for the three participants with either predominantly slow-twitch or fast-twitch muscle fibre typology.

Only the individuals with primarily type I muscle fibre typology were able to enhance high-intensity running performance (Table 1). For those individuals, there was an increase in the number of 19 s running bouts [Cohen’s d: 2.75 (large)] resulting in an increase in total running distance (with the distance during recovery) [Cohen’s d: 4.05 (large)] and high-intensity running distance (without the distance during recovery) [Cohen’s d: 3.21 (large)] by 17 ± 12% and 15 ± 11%, respectively. There were no differences between the groups regarding changes in heart rate and lactate at exhaustion.

## 3. Discussion

The present exploratory study provided evidence that the inter-individual responses of the enhanced performance of high-intensity intermittent treadmill running due to the intake of anthocyanin-rich New Zealand blackcurrant may be related to the individual muscle fibre typology of the *m. quadriceps femoris*. A limitation of our exploratory study was that muscle fibre typology was not determined by gold-standard biopsy information. Muscle fibre typology was indirectly based on a performance study with repeated maximal isometric contractions by Hamada et al. [17] and biopsy observations from Hamada et al. [18]. We were able to replicate the findings from Hamada et al. [17] regarding the isometric fatigue profile of the *m. quadriceps femoris*, allowing us to identify individuals with a type I or type II muscle fibre typology. According to the information in Hamada et al. [17], the individuals (*n* = 4) with type II muscle fibre typology had an isometric force decline of ~48% and this was comparable with our observation of 46%. Our participants that were categorized as having a predominance of type I muscle fibres had an isometric force decline of 12% and this was less than the value of ~21% in Hamada et al. [17]. In Hamada et al. [17], no information was provided on verbal encouragement during fatigue testing, training status, and activity level of the participants, as these are all factors that can contribute to the exercise-induced fatigue profile of individuals.

In the present exploratory study, the three individuals that were classified as having predominant type I muscle fibres in the *m. quadriceps femoris* experienced enhanced performance during high-intensity intermittent treadmill running with the intake of New Zealand blackcurrant extract. For the three individuals that were classified as having predominantly type II muscle fibres, there was no, or even a negative, ergogenic effect on the performance of high-intensity treadmill running. The lack of significant correlations between the force decline from the repeated isometric contractions and the change in the total running and high-intensity running distance is likely due to most individuals having a mixed muscle fibre typology that does not allow them to be categorized as primarily type I or II. Our observations of a beneficial effect in individuals with a type I muscle fibre typology may seem counterintuitive, with respect to the ergogenic effects of anthocyanin-rich blackcurrant extract, as an improvement in high-intensity running performance would be more likely to be expected with postponing the peripheral fatigue mechanisms in type II muscle fibres. It is possible that both muscle fibre types experience different levels of oxidative stress-induced fatigue [38], and that the antioxidant effects of anthocyanins and anthocyanin-induced metabolites may meaningfully affect the type I muscle fibres and not the type II muscle fibres. Conclusive studies on exercise-induced and muscle fibre-type dependent redox state alterations are lacking. In addition, it would be of interest to examine the potential of other polyphenol-containing supplementation and whether there would be muscle fibre-type specific effects on the high-intensity exercise responses (e.g., lychee extract [39]) and recovery from high-intensity intermittent exercise (e.g., Montmorency tart cherry [40]). It needs to be noted that the mechanisms for the performance enhancing effects of the intake of anthocyanin-rich blackcurrant are still unknown. Studies have shown performance enhancing effects in different exercise modalities, including endurance exercise of 16.1 km cycling time trial performance [41], and 5 km treadmill running [42], as well as high-intensity exercise and maximal sprints [1,2,3]. The mechanisms that enhance the performance of exercise with different intensities and duration are not similar. Therefore, our observations should not be generalized to the idea that exercise performance enhancement due to the intake of anthocyanin-rich blackcurrant is due to primarily affecting the fatigue profile of type I muscle fibres. We can also not exclude that the muscle-fibre type dependence was unique for the training status of our cohort of healthy recreationally active men. However, in a study that examined sex differences in response to the intake of sodium bicarbonate with muscle fibre type suggested to contribute to the differences in Wingate and throwing performance, the cohort consisted of members of the Polish national wrestling team [34]. It is possible that the ergogenic potential of blackcurrant extract to affect high-intensity intermittent running performance shows muscle fibre-type dependence for individuals with differences in physical training status. Nevertheless, future work is required to examine in single slow-twitch and fast-twitch muscle fibres the effect of anthocyanin-induced metabolites on muscle fibre fatigue characteristics. Single muscle fibre work is available for other compounds, e.g., piperine [43]. Piperine was able to change the contractile responses for slow- and fast-twitch muscle fibres, e.g., increases in low-frequency force, the rate of force development and relaxation rate, but there was no muscle-fibre type dependence [43]. Future single muscle fibres studies will complement existing studies on the beneficial effects of anthocyanin-rich blackcurrant on whole-body exercise performance. Whether muscle fibre type and nutritional ergogenic potential is a consideration for personalized sports nutrition strategies is still unclear. It needs to be acknowledged that we did not control for the habitual polyphenol intake that could affect composition of the gut microbiome, plasma bioavailability of metabolites, and the responses for participants with type I and type II typology. Interindividual variation in metabolites occurs [44] and requires an intact gut microbiome [45].

## 4. Materials and Methods

### 4.1. Participants and Experimental Design

For this exploratory study, healthy recreationally active men (*n* = 16, Caucasian, age: 23 ± 3 years, body mass: 79 ± 11 kg, height: 179 ± 5 cm) volunteered. Sample size was based on a previous study examining the effects of NZBC extract on high-intensity running performance [1]. Participants were current participants in sport/training activities or habitual exercise with high-intensity, intermittent running. Participants had no known allergy to anthocyanin-containing foods or products and they were not allowed to take other dietary supplements during the study. The study was approved by the University of Chichester Research Ethics Committee (approval date: 21 June 2016, approval code: 2425_12). Methodological procedures of the study adhered to the guidelines of the Declaration of Helsinki by the World Medical Association. Participants provided written informed consent after receiving written information about the objectives, the experimental procedures, and the requirements of participation in the study. The study had a placebo-controlled, randomized, cross-over design. The main objective of this exploratory study was to identify participants with a predominant type I or type II muscle fibre typology using an in vivo fatiguing exercise protocol with repeated voluntary maximal contraction of the *m. quadriceps femoris* by Hamada et al. [17]. Fibre type was inferred indirectly via isometric fatigue and not by muscle biopsy, and the fatigue profile may be influenced by training status, motivation, or other neuromuscular factors. For the participants with a predominant type I or type II muscle fibre typology, the performance responses with intake of New Zealand blackcurrant extract on high-intensity intermittent running protocol were examined. Note that the data for the cohort and inter-individual responses to repeated testing were reported elsewhere [29].

### 4.2. Fatigue Testing of the Quadriceps Femoris Muscles with Isometric Contractions

The participants were positioned on a custom-built chair. One Velcro strap over the chest and one over the belly were used to hold the upper body firmly against the back of the chair. The participants were required to keep the arms crossed across their chest and instructed to keep the upper body straight and not lean forward during the force recordings measurements of the *m. quadriceps femoris*. A custom-made metal clamp was positioned around the lower right leg above the medial malleolus with a polyester strap. The metal clamp was connected with a steel chain to allow for the connection of the ankle with a calibrated s-beam load cell (RS 250 kg, Tedea Huntleigh, Cardiff, UK). Sampling frequency of the force recordings of the *m. quadriceps femoris* was 1000 Hz (PowerLab data acquisition system, ADinstruments Ltd., Oxford, UK). The force recordings were displayed on a computer screen [(Chart for Windows (Chart 4 V4.1.2, AD Instruments, Oxford, UK)] that was positioned about 1.5 m in front of the participants. All participants practiced maximal isometric voluntary contractions and the fatiguing exercise protocol with repeated maximal isometric voluntary contractions. In brief, the fatiguing exercise protocol by Hamada et al. [17] consists of 16 iMVCs of 5 s each with a 3 s rest period.

### 4.3. Baseline and Experimental Testing

The exploratory study had 5 visits over a period of 12 to 16 weeks. All testing sessions were conducted in an exercise physiology laboratory (17–19 °C and 60–75% humidity) with the running tests carried out on a motorized treadmill (H/P/COSMOS, Groningen, The Netherlands) at a 1% gradient. In the first visit, maximum oxygen uptake ($\dot{V}$O_2max_) was determined with a rapid ramp test to exhaustion and was followed after 10 min with a verification phase test to confirm $\dot{V}$O_2max_ [46]. The rapid ramp test commenced at an individually determined speed and increased by 0.1 km·h^−1^ every 5 s until exhaustion. The verification square wave test commenced with a 3 min period at 50% of the speed at $\dot{V}$O_2max_, before an abrupt increase to 100% of the speed at $\dot{V}$O_2max_. During both tests, no feedback was provided but strong verbal encouragement was provided to motivate participants to reach volitional exhaustion. The $\dot{V}$O_2max_ and speed at $\dot{V}$O_2max_ determined with the rapid ramp test to exhaustion were 55.3 ± 5.0 mL·kg^−1^·min^−1^ and 17.2 ± 0.8 km·h^−1^. Then, the participants were familiarized with the high-intensity, intermittent treadmill running test. The protocol will be described below. In the four subsequent visits (i.e., visits 2 to 5), each participant completed two placebo and two NZBC extract trials. The running protocol was used by Perkins et al. [1,29] and involved three phases after standardized warm-up. The first phase consisted of 5 min running at 60% of the speed at $\dot{V}$O_2max_ and the speed was 13.8 ± 0.6 km·h^−1^. The second phase had 7 stages. Each stage in the second phase lasted 204 s and consisted of six repeated high-intensity running bouts lasting 19 s with active recovery bouts at 50% of the speed at $\dot{V}$O_2max_ (speed: 8.6 ± 0.4 km·h^−1^) that lasted 15 s. Between each stage in the second phase, there was a passive recovery of 1 min in which a fingertip blood sample was collected for lactate. The speed for the high-intensity running bouts was calculated by a percentage of the speed at $\dot{V}$O_2max_, with stage one being set at 80% of the speed at $\dot{V}$O_2max_. Running speed of the high-intensity running bouts in each stage was then increased by 5% of the speed at $\dot{V}$O_2max_ (0.9 ± 0.0 km·h^−1^) per stage, up to 110% of the speed at $\dot{V}$O_2max_ (stage 6). After stage 7 in the second phase, phase three (≥stage 7) was initiated with speed increases by 2.5% of the speed at $\dot{V}$O_2max_ (0.4 ± 0.0 km·h^−1^) per stage, with similar running times as in phase two. Note that exhaustion could be reached in the second or third phase. Treadmill settings allowed ~2–4 s for acceleration/deceleration between speeds depending on the required change in velocity. During the test, participants were informed of the beginning and end of a high-intensity running bout but were not provided with information on bout and stage number. Strong verbal encouragement was also provided. On reaching voluntary exhaustion, the protocol allowed the measurement of total running distance on the treadmill (including the recovery distance) and the distance covered during the high-intensity running bouts (without the recovery distance). Volumes and analysis of expired air were measured via a breath-by-breath gas analyser (Jaegar Oxycon Pro, Cardinal Health, Basingstoke, UK). The analyser was calibrated with gases of known concentration, and the tube flowmeter was calibrated by a 3 L syringe. Blood samples at exhaustion for lactate were analyzed within 30 s of collection (YSI 2300, Analytical Technologies, Farnborough, Hants, UK). Heart rate (Consultancy RS800, Polar Electro UK Ltd., Warwick, UK) was recorded during each exercise protocol. Participants recorded their food intake and physical activity in the 48 h preceding the first experimental visit and were asked to replicate this in the 48 h preceding the subsequent visit. Participants refrained from caffeine and alcohol 24 h preceding each session and abstained from vigorous exercise during this period. All testing was conducted in the morning (±2 h).

### 4.4. Supplementation

The randomized dosing strategy for the present study included a capsulated NZBC extract or placebo for 7 days. NZBC extract supplementation was provided two times for seven days with 210 mg anthocyanin per day in a 600 mg extract dose with two capsules per day. According to company information, each capsule contained delphinidin-3-rutinoside (35–50%), delphinidin-3-glucoside (5–20%), cyanidin-3-rutinoside (30–45%), and cyanidin-3-glucoside (3–10%) (CurraN^TM^, Health Currancy Ltd., Surrey, UK; CurraNZ Ltd., Auckland, New Zealand). The placebo was administered two times for seven days (600 mg microcrystalline cellulose M102 in two match-coloured capsules per day). On the morning of the seventh day of intake, the final two capsules were consumed two hours prior to testing. On testing days, participants were instructed to arrive in a fully hydrated state and to consume a slice of toast without butter ≥2 h prior to arrival. The experimental visits were separated by a period of at least 21 days and no more than 45 days. This would allow at least a 14-day wash-out period for the NZBC extract before subsequent intake of either NZBC extract or placebo. No objective information on adherence to the intake was recorded for extract and placebo supplementation.

### 4.5. Statistical Analysis

Graphpad Prism (version 5.00 for Windows, GraphPad Software, San Diego, CA, USA) was used for statistical analyses. Unpaired samples t-test was used for analysis of the type I (*n* = 3) and type II (*n* = 3) observations. It needs to be noted that we did not perform a power calculation to justify the small number of participants with type I and Type II fatigue profile. The coincidental similarity for the participants with the three lowest and three highest fatigue profile with those in Hamada et al. [17] was taken to analyze the data for potential type I and type II ergogenic blackcurrant effects. Correlation was quantified and analyzed for the cohort relationship between the isometric force decline and the change in total running distance as well as the high-intensity running distance. Data are reported as mean ± SD. Statistical significance was set at *p* < 0.05. Cohen’s *d* effect sizes were calculated for parameters with significant changes and considered trivial (*d* < 0.2), small (*d* = 0.2–0.49), moderate (*d* = 0.5–0.79) or large (*d* ≥ 0.8)

## 5. Conclusions

From the present exploratory study, the findings suggest that the ergogenic potential of anthocyanin-rich blackcurrant extract to enhance high-intensity exercise performance may depend on muscle fibre type. Based on the fatigue profile during a series of voluntary maximal isometric contractions of the *m. quadriceps femoris*, individuals that were classified as having predominant type I muscle fibres in the *m. quadriceps femoris* experienced enhanced performance during high-intensity intermittent treadmill running with intake of New Zealand blackcurrant extract. Our observations contribute to our understanding of the underpinning of the interindividual responses to high-intensity exercise by the intake of anthocyanin-rich New Zealand blackcurrant extract. Future work should address how anthocyanin-induced metabolites affect the peripheral fatigue mechanism in type I and type II muscle fibres.

## Figures and Tables

**Table 1:** Number of high-intensity running bouts of 19 s, running distances, heart rate, and lactate at exhaustion of the high-intensity intermittent running test for three participants with predominant type I and type II muscle fibre typology. #, number; TRD, total running distance; HIRD, high-intensity running distance; ∆TRD, change in total running distance between NZBC and placebo conditions; ∆HIRD, change in high-intensity running distance between NZBC and placebo conditions; NZBC, New Zealand blackcurrant.

	Type I Muscle Fibre	Type II Muscle Fibre	*p*-Value
# running bouts_placebo_	31 ± 8	35 ± 4	0.487
∆# running bouts	8 ± 4	−3 ± 4	0.030
			
TRD_placebo_ (m)	3756 ± 1390	4270 ± 473	0.577
∆TRD (m)	526 ± 193	−245 ± 188	0.008
			
HIRD_placebo_ (m)	2416 ± 898	2712 ± 288	0.616
∆HIRD (m)	308 ± 110	−136 ± 162	0.017
			
Heart rate_placebo_ (bpm)	196 ± 7	186 ± 7	0.156
∆Heart rate (bpm)	0 ± 1	1 ± 4	0.792
			
Lactate_placebo_ (mmol·L^−1^)	4.57 ± 1.05	4.41 ± 0.84	0.854
∆Lactate (mmol·L^−1^)	0.47 ± 0.92	0.90 ± 1.13	0.632

## Data Availability

The raw data supporting the conclusions of this article will be made available by the authors on request.

## Abbreviations

The following abbreviations are used in this manuscript:

iMVC	isometric maximal voluntary contraction
HIRD	high-intensity running distance
NZBC	New Zealand blackcurrant
TRD	total running distance
$\dot{V}$O_2max_	maximum oxygen uptake
